# Plasma Volume Expansion Resulting from Intravenous Glucose Tolerance Test

**DOI:** 10.1155/2011/965075

**Published:** 2011-09-28

**Authors:** Robert G. Hahn, Thomas Nyström

**Affiliations:** ^1^Section for Anaesthesia, Faculty of Health Sciences, Linköping University, 58185 Linköping, Sweden; ^2^Research Unit, Södertälje Hospital, 152 86 Södertälje, Sweden; ^3^Department of Clinical Science and Education, Karolinska Institutet, Södersjukhuset, 11883 Stockholm, Sweden

## Abstract

*Objective*. To quantify the degree of plasma volume expansion that occurs during an intravenous glucose tolerance test (IVGTT). *Methods*. Twenty healthy volunteers (mean age, 28 years) underwent IVGTTs in which 0.3 g/kg of glucose 30% was injected as a bolus over 1 min. Twelve blood samples were collected over 75 min. The plasma glucose and blood hemoglobin concentrations were used to calculate the volume distribution (*V*_*d*_) and the clearance (*CL*) of both the exogenous glucose and the injected fluid volume. *Results*. The IVGTT caused a virtually instant plasma volume expansion of 10%. The half-life of the glucose averaged 15 min and the plasma volume expansion 16 min. Correction of the fluid kinetic model for osmotic effects after injection reduced *CL* for the infused volume by 85%, which illustrates the strength of osmosis in allocating fluid back to the intracellular fluid space. Simulations indicated that plasma volume expansion can be reduced to 60% by increasing the injection time from 1 to 5 min and reducing the glucose load from 0.3 to 0.2 g/kg. *Conclusion*. A regular IVGTT induced an acute plasma volume expansion that peaked at 10% despite the fact that only 50–80 mL of fluid were administered.

## 1. Introduction

The intravenous glucose tolerance test (IVGTT) is a tool for assessment of glucose disposal in diabetes research and clinical investigations. The test includes a bolus injection of hypertonic glucose and repeated measurement of the plasma glucose and insulin concentrations over up to 3 hours, sometimes with administration of insulin after 20 min [1, 2]. Several important indices can be derived from an IVGTT, including insulin sensitivity and beta cell function [3]. 

An overlooked aspect of IVGTTs is the cardiovascular strain caused by osmotic translocation of intracellular fluid caused by hypertonic glucose. Only 50–80 mL of fluid are administered, but how much and how fast the plasma volume becomes expanded does not seem to have been previously studied. This is of interest, as the test might be administered to diabetic patients with limited cardiovascular reserves. 

Mathematical methods for analysis and simulation of the disposition of hypertonic crystalloid [4, 5] and isotonic glucose solutions [6, 7] have been developed during the past decade. In the present study, we combine these methods to analyze the degree and time course of the hypervolemic effect of IVGTTs in 20 healthy volunteers.

## 2. Subjects and Methods

Twenty volunteers, 8 females and 12 males, aged between 18 and 51 years (mean, 28) and with a body weight of 49–88 kg (mean, 68) were studied. The study was approved by the Regional Ethics Committee of Stockholm, and informed consent was obtained from the participants. Mean BMI was 23.4 (SD 2.3) kg/m^2^, and the serum concentrations of electrolytes and of HbA1c were normal in all cases. None of the subjects used daily medication.

### 2.1. Procedure

The experiments were conducted at the Department of Endocrinology at Södersjukhuset and began at approximately 8 am. After an overnight fast, each volunteer was placed comfortably on a bed. In each subject, a cannula was inserted into the antecubital vein of each arm, one for sampling blood and the other for infusing fluid. Monitoring consisted of electrocardiography, pulse oximetry, and noninvasive blood pressure. 

After a 30 min equilibration period to obtain hemodynamic steady state, a short regular IVGTT was performed by administrating 0.3 g/kg of glucose in a 30% solution over 1 min. Blood was sampled at 0, 2, 4, 6, 8, 10, 20, 30, 40, 50, 60, and 75 min for assessment of the plasma glucose and blood hemoglobin (Hb) concentrations. Plasma glucose was measured by the glucose oxidase method used by the hospital's usual laboratory, and the Hb concentration was measured by colorimetry (Technicon Advia, Bayer, Tarrytown, NY, USA).

### 2.2. Pharmacokinetics

#### 2.2.1. Glucose

Plasma concentration *G* at time *t* when glucose was infused at rate *R*_0_ was calculated using the following differential equation:

(1) $$\frac{d\left(G-G_{b}\right)}{dt}=\frac{R_{0}}{V_{d}}-\frac{CL}{V_{d}}*\left(G\left(t\right)-G_{b}\right),$$

where *G*_*b*_ is the baseline glucose, *V*_*d*_ is the volume of distribution, and *CL* is the clearance. Since glucose enters the cells by active transport, a decreasing amount of glucose in *V*_*d*_ corresponds, in the absence of glucosuria, to the uptake of glucose into cells [6, 7].

The half-life (*T*_1/2_) of the exogenous glucose load was obtained as [ln 2∗*V*_*d*_/*CL*].

#### 2.2.2. Fluid

Hypertonic glucose causes an osmotic shift that draws water from the intracellular (40% of the body weight (BW)) to the extracellular (20% of the BW) fluid space. Using the baseline serum osmolality (295 mosmol/kg), the translocated volume *f*_*t*_ resulting from each injection was obtained as follows [4]:

(2) $$\frac{\text{BW}*20\text{\%}\cdot 295+\text{infused}\;\text{osmoles}}{\text{BW}\cdot 20\text{\%}+f_{t}+\text{infused}\;\text{volume}}=\frac{\text{BW}\cdot 40\text{\%}\cdot 295}{\text{BW}\cdot 40\text{\%}-f_{t}}.$$

The kinetic model calculates the baseline volume of distribution (*V*) and the clearance (*CL*) for the sum of the injected (*R*_0_) and translocated (*f*_*t*_) fluid volumes. The model implies that the fluid added to the kinetic system expands *V* to *v*, which strives to return to *V* by two mechanisms: first, elimination of fluid at a rate proportional by a constant *CL* to the dilution of *V*, and second, a baseline loss (*CL*_0_) fixed to 0.4 mL/min to account for evaporation and basal diuresis [4]. The volume change of *v* is then expressed as

(3) $$\frac{dv}{dt}=R_{0}+f_{t}-CL_{0}-CL\frac{\left(v-V\right)}{V}.$$

The dilution of *v*, which is given by (*v* − *V*)/*V*, was set equal to the plasma dilution as derived from the blood Hb concentration at baseline time 0 and at time *t*. Hence, [(Hb_0_/Hb(*t*))  –  1]/(1  –  hematocrit_0_).

The optimal estimates for the unknown parameters in the glucose and fluid models were calculated for each of the 20 experiments individually by nonlinear least-squares regression. No weights were used. The software used was Matlab 4.2 (Math Works Inc., Natick, Mass, USA). 

The half-life (*T*_1/2_) of the infused fluid volume was obtained as [ln 2∗*V*/*CL*]. The result of the kinetic analysis of glucose has been published elsewhere [8].

### 2.3. Statistics

The results were presented as mean and standard deviation (SD) and, when there was a skewed distribution, as the median (25th–75th percentile range). All reported correlations were statistically significant by *P* < 0.05.

## 3. Results

Baseline plasma glucose was 4.8 (0.5) mmol/L, and the blood Hb concentration was 13.5 (11.7–14.0) g/dL. 

**Figure 1:**
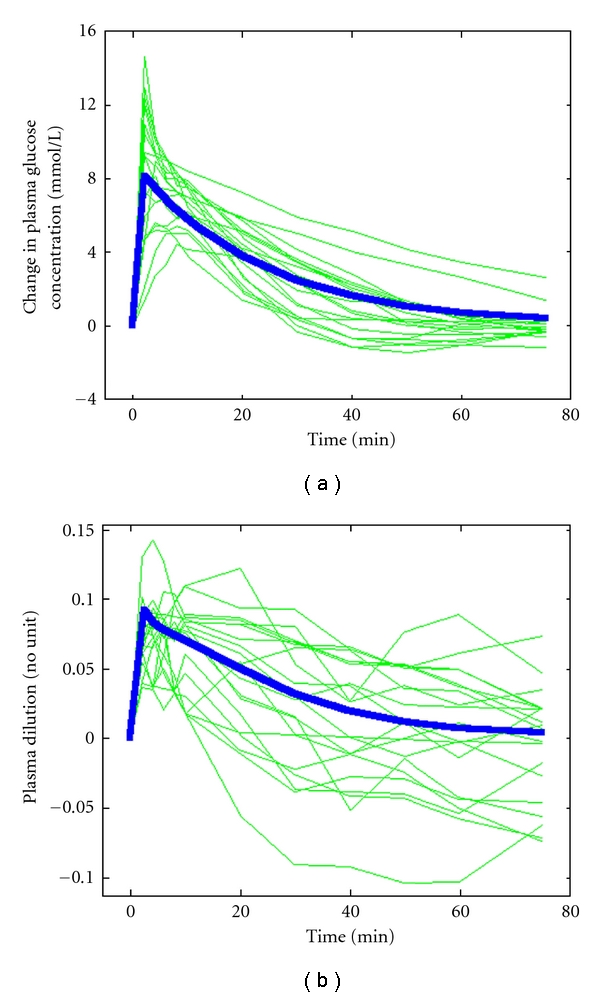
(a) Change in plasma glucose concentration. (b) Dilution of venous plasma calculated from changes in the blood hemoglobin concentration in response to an intravenous injection of 0.3 g/kg of glucose over 1 min. Each experiment is represented by a thin line and the modeled average by a thick line.

**Figure 2:**
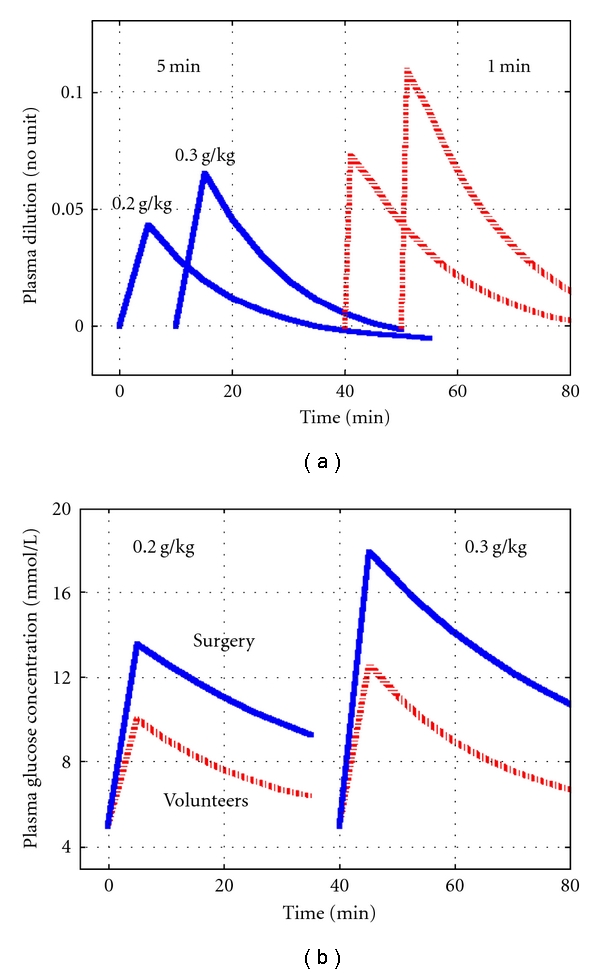
(a) Simulation showing the plasma volume expansion that would ensue if the injection time was increased to 5 min (left) from 1 min (right) for an IVGTT dose of 0.2 g/kg and 0.3 g/kg of glucose, respectively. (b) Simulation of the plasma glucose level if the glucose load was decreased to 0.2 g/kg (left) from 0.3 g/kg (right) in surgical patients (solid line) and in volunteers (broken line). The kinetic data for volunteers were taken from the present study, and those for surgery (*V*_*d*_  =  9.1 L and *CL*  =  0.21 L/min) were derived from [7].

All 20 experiments could be analyzed with the proposed equations for plasma glucose and insulin kinetics. The modeled rise in plasma glucose was 8 mmol/L (Figure 1(a)), and the plasma volume expansion, as evidenced by the plasma dilution, was 9% at 2 min (Figure 1(b)). Simulation showed that the peak would be almost 11% at the end of the 1 min injection (Figure 2(a)). 

### 3.1. Kinetic Parameters

The *V*_*d*_ and *CL* for the administered glucose were 14.0 (6.5) L and 0.63 (0.26) L/min, respectively [8].

Calculation of the osmotic fluid shift resulting from the injection of hypertonic glucose showed that each mmol of exogenous glucose translocated 1.83 mL of fluid from the intra- to the extracellular fluid space. The translocated plus injected fluid volume expanded a body fluid space of 3.0 (2.5–4.0) L in size, and the *CL* was 0.13 (0.04–0.44) L/min. The half-lives of the exogenous glucose and the fluid volume was similar, at 15 (12–19) min and 16 (6–44) min, respectively. However, the variability between the subjects was greater for the fluid component (cf. Figures 1(a) and 1(b)). 

Recalculating the fluid kinetics on the assumption that 1.83 mL of fluid was eliminated for each mmol of eliminated glucose yielded practically the same size *V* but a fluid *CL* of only 0.02 L/min (median), that is, almost all of the disappearance of fluid could be attributed to osmosis.

### 3.2. Simulations

Computer simulations were performed based on the average parameters obtained by the kinetic analysis of the glucose and fluid kinetics. 

The simulations suggested that the brisk peak in plasma dilution at the end of the injection of hypertonic glucose could be reduced to 60% by lowering the dose of glucose from 0.3 to 0.2 g/kg and extending the time of injection from 1 to 5 min (Figure 2(a)). 

Modification of the IVGTT could also be justified in clinical situations associated with altered glucose kinetics. Simulations using *V*_*d*_  =  9.1 L and *CL*  =  0.21 L/min as derived from a previous study of laparoscopic cholecystectomy [7] indicated that the conventional dose for an IVGTT of 0.3 g/kg would, in this clinical setting, raise plasma glucose to >12 mmol/L during as long a time as 30 min, during which glucosuria could ensue. In contrast, the lower dose, 0.2 g/kg, would exceed this limit only during 10 min (Figure 2(b)).

## 4. Discussion

The hypertonic glucose caused a virtually instant plasma volume expansion of 10%, which corresponded to approximately 300 mL in our volunteers. Most of this volume (75%) was allocated from the intracellular fluid space by virtue of osmosis. The return of the plasma volume to normal was also governed by osmosis, as *CL* for the fluid became almost nil when the kinetic model was corrected for the redistribution of water driven by the uptake of glucose to the cells. 

Volume expansion was much greater than would be expected if assuming that fluid translocated by osmosis is distributed throughout the extracellular fluid space. If that had been the case, the plasma volume expansion of 10% would have represented an extracellular volume expansion of almost 1.4 L, which is unreasonable considering the limited amount of injected osmotically active glucose molecules. Rather, mass balance calculations indicate that the translocated plus injected fluid volume was in the range of 300 mL. Hence, the size of the body fluid space expanded by the infused volume could not have been larger than the plasma volume. This finding is not intuitive but is in agreement with previous kinetic studies based on IV infusion of isotonic glucose solutions in healthy volunteers [6, 9] and in surgical [7] and diabetic [10] patients.

Plasma dilution of 10% is not completely trivial, as it corresponds to that measured at the end of a 45 min infusion of 12.5 mL/kg (approximately 1 L) of glucose 2.5% with electrolytes [9]. Moreover, plasma volume expansion during an IVGTT develops so quickly that the circulatory system cannot adapt gradually to the situation. In our study, the rapid onset even made it difficult to capture the peak. As Hb was sampled 1 min after the end of the injection, the curve in Figure 1(b) indicated a plasma dilution of only 9%. On the other hand, the computer simulation shown in Figure 2(a) indicates that the maximum dilution at the very end of the injection would be even somewhat higher than 10% (Figure 2(a)). However, this value is theoretical, as the circulation time is approximately 1 min. 

The degree and speed of the onset of plasma volume expansion would be far less dramatic by decreasing the dose of hypertonic glucose from 0.3 to 0.2 g/kg and by increasing the injection from 1-2 minutes to 5 minutes. The simulations we performed based on the kinetic parameters suggest that the plasma volume expansion would then be cut in half and also develop more slowly. 

The kinetic analyses also argue against the use of a conventional IVGTT in the presence of surgery. A smaller dose, 0.2 g/kg, would smooth the hyperglycemia enough to prevent prolonged glycosuria (*≈*30 min) in this setting. The kinetics for the doses of glucose simulated here are known to be linear; that is, the same values of *V*_*d*_ and *CL* are obtained when the dose and infusion time are changed [6]. 

A limitation of the present study is that no invasive hemodynamic monitoring was performed. However, one would hardly expect problems in the cardiovascular adaptation to the rapid onset of plasma volume expansion in the group of healthy volunteers used in this study. Hemodynamic measurements would be of greater interest in elderly subjects with reduced cardiovascular reserves, such as diabetic patients. Insulin-resistant persons, such as those with obesity, would also be likely to experience a more long-lasting plasma volume expansion with an ensuing risk of hemodynamic stress. The reason is that the fluid clearance is governed almost completely by the rate of uptake of glucose to the cells, which occurs more slowly in insulin-resistant states.

In conclusion, the plasma volume expansion resulting from an IVGTT is greater than commonly believed (10%) because the injected and osmotically translocated fluid have a volume of distribution that corresponds only to the plasma volume instead of to the entire extracellular fluid space. A smaller dose and slower injection time might be appropriate if an IVGTT is applied in debilitated or surgical patients.
